# Gender equality and human rights approaches to female genital mutilation: a review of international human rights norms and standards

**DOI:** 10.1186/s12978-017-0322-5

**Published:** 2017-05-12

**Authors:** Rajat Khosla, Joya Banerjee, Doris Chou, Lale Say, Susana T. Fried

**Affiliations:** 1 UNDP/UNFPA/UNICEF/WHO/World Bank Special Programme of Research, Development and Research Training in Human Reproduction (HRP), Department of Reproductive Health and Research, World Health Organization, Headquarters, 20 Avenue Appia, Geneva, 1211 Switzerland; 2 Jhpiego, an affiliate of Johns Hopkins University, Gender Technical Advisor, 1776 Massachusetts Avenue, NW, Suite 300, Washington DC, 20036 USA; 3 Fellow, Global Health Justice Partnership, Yale University, 170 15th St., New York, NY 11215 USA; 4 Department of Reproductive Health and Research, World Health Organization, 20, Avenue Appia, CH-1211 Geneva 27, Switzerland

**Keywords:** Human rights, Gender equality, Gender equity, Gender discrimination, Female genital mutilation, Female genital cutting, Female circumcision, Gender norms, Harmful traditional practices, Violence against women, Gender-based violence

## Abstract

Two hundred million girls and women in the world are estimated to have undergone female genital mutilation (FGM), and another 15 million girls are at risk of experiencing it by 2020 in high prevalence countries (UNICEF, 2016. Female genital mutilation/cutting: a global concern. 2016). Despite decades of concerted efforts to eradicate or abandon the practice, and the increased need for clear guidance on the treatment and care of women who have undergone FGM, present efforts have not yet been able to effectively curb the number of women and girls subjected to this practice (UNICEF. Female genital mutilation/cutting: a statistical overview and exploration of the dynamics of change. 2013), nor are they sufficient to respond to health needs of millions of women and girls living with FGM. International efforts to address FGM have thus far focused primarily on preventing the practice, with less attention to treating associated health complications, caring for survivors, and engaging health care providers as key stakeholders. Recognizing this imperative, WHO developed guidelines on management of health complications of FGM. In this paper, based on foundational research for the development of WHO’s guidelines, we situate the practice of FGM as a rights violation in the context of international and national policy and efforts, and explore the role of health providers in upholding health-related human rights of women at girls who are survivors, or who are at risk. Findings are based on a literature review of relevant international human rights treaties and UN Treaty Monitoring Bodies.

## Plain English Summary

Two hundred million girls and women are estimated to have undergone female genital mutilation (FGM) [1], a traditional practice that involves the partial or total removal of the external genitalia. FGM is a dire violation of human rights— particularly women and children’s rights— and results in severe health complications, including but not limited to death, disability, miscarriage, stillbirth, shock, hemorrhage, sepsis, sexual dysfunction and post-traumatic stress disorder. Although the practice is not sanctioned by any religion and is illegal in many countries, it is prevalent in 30 African countries, a few in Asia and the Middle East, and, due to international migration, across the globe. Although the prevalence or support for the practice has decreased in some countries, in others, it has reportedly increased or stayed the same [2].

International efforts to address FGM have thus far focused primarily on preventing the practice, with less attention to treating associated health complications, caring for survivors, and engaging health care providers as key stakeholders who can help in the abandonment of the practice. Few pre or in-service training programs for health providers address how to recognize and treat FGM, and there are fewer tools and programs to provide healthcare to women who have undergone FGM, compared with those that prevent it from ever occurring. In addition, health providers are often reluctant to address the topic because they may feel they have no role in addressing cultural practices.

This led the World Health Organization to develop a set of guidelines for health providers to care for women living with FGM. This paper was commissioned as part of the development of these guidelines to ensure that health providers understand international policy and the human rights basis for upholding women and children’s human rights when it comes to FGM, especially their duty to never perform the procedure, to refuse requests to re-perform the procedure after childbirth, to prevent it from continuing, and to safeguard the rights of women and girls living with FGM.

## Background

Two hundred million girls and women in the world are estimated to have undergone female genital mutilation (FGM) [1] and another 3 million girls are at risk of experiencing it each year in high prevalence countries [2]. FGM has been reported in all parts of the world, but it is most prevalent in the western, eastern, and north-eastern regions of Africa, some countries in Asia and the Middle East and among several immigrant communities in North America, Europe and Australia [3]. Many contextual factors stemming from gender inequality have been documented to perpetuate FGM, for example: highly unequal societies in which gender prescriptions demand girls’ virginity prior to marriage, [4–6] women’s chastity and monogamy in marriage, [4, 7, 8] sexual availability of females to their male partners, [9] and the production of legitimate male heirs to further their husband’s patrilineage [5, 6]. Other motivations include concerns about girls’ marriageability and social acceptance, and the fear of a loss of protection by other women and the community at large if a girl does not undergo FGM [5, 10–14].

Despite decades of efforts to eradicate or abandon the practice, and the increased need for clear guidance on the treatment and care of women who have undergone FGM, present efforts have not yet been able to effectively curb the number of women and girls subjected to this practice [2], nor are they sufficient to respond to health needs of millions of women and girls living with FGM. International efforts to address FGM have thus far focused primarily on preventing the practice, with less attention to treating associated health complications, caring for survivors, and engaging health care providers as key stakeholders. The WHO Guidelines Development Group reviewed existing guidance and peer-reviewed literature on FGM programs and resources, and found that the majority of it focused on prevention. Few pre - or in-service training programs for health providers address how to recognize and treat FGM in a respectful and non-judgmental manner, and there are far fewer tools and programs to provide healthcare to women who have undergone FGM, compared with those that prevent it from ever occurring. In recent years, several governments have criminalized the practice, with mixed results, but prompting renewed attention to the issue.

Recognizing this imperative, WHO developed the 2016 Guidelines on the Management of Health Complications from FGM [15]. This paper is based on background research that was conducted to inform the development of the WHO Guidelines and to contextualize the guidelines within the human rights dimension of FGM and health. Other ongoing attention by the United Nations (UN) to this issue includes, for example, the inclusion of a relevant target in the Sustainable Development Goals, [16] and the UN Secretary General’s Global Strategy on Women’s, Children’s and Adolescents Health [17] and its programmatic work at the UN agency level including, in particular, a joint UNICEF and UNFPA programme on FGM) [18].

The guiding principles (Table 1), recommendations and best practice statements of the WHO Guidelines (Table 2) were informed by a review of international human rights norms and standards [15], that explored the role of health providers in upholding health-related human rights of women at girls who are survivors or who are at risk of undergoing FGM. The Guidelines were developed to respond to the role of “health-care providers across the globe, many of whom have received little or no formal education on the issue of FGM, may find themselves ill-prepared to make sensitive enquiries about FGM and to treat and care for girls and women with FGM-related complications.” The guidelines address certain questions faced by health providers treating women and girls who have undergone FGM, which require an understanding of the human rights dimensions, such as what counseling and care should be provided, what to do if they suspect a girl is at risk, how to handle requests by a woman or family members to reinfibulate a woman after delivery, and whether or not it is the role of the health provider to counsel families against the practice.

**Table 1:** Guiding Principles [15]

I. Girls and women living with female genital mutilation (FGM) have experienced a harmful practice and should be provided quality health care. II. All stakeholders – at the community, national, regional and international level – should initiate or continue actions directed towards primary prevention of FGM. III. Medicalization of FGM (i.e. performance of FGM by health-care providers) is never acceptable because this violates medical ethics, since (i) FGM is a harmful practice; (ii) medicalization perpetuates FGM; and (iii) the risks of the procedure outweigh any perceived benefit.

**Table 2:** Summary of the Recommendations and Best Practice Statements [15]

Deinfibulation
R-1. Deinfibulation is recommended for preventing and treating obstetric complications in women living with type III FGM
R-2. Either antepartum or intrapartum deinfibulation is recommended to facilitate childbirth in women living with type III FGM
R-3. Deinfibulation is recommended for preventing and treating urologic complications – specifically recurrent urinary tract infections and urinary retention – in girls and women living with type III FGM
BP-1. Girls and women who are candidates for deinfibulation should receive adequate preoperative briefing
BP-2. Girls and women undergoing deinfibulation should be offered local anaesthesia
Mental health
R-4. Cognitive behavioural therapy (CBT) should be considered for girls and women living with FGM who are experiencing symptoms consistent with anxiety disorders, depression or post-traumatic stress disorder (PTSD)
BP-3. Psychological support should be available for girls and women who will receive or have received any surgical intervention to correct health complications of FGM
Female sexual health
R-5. Sexual counselling is recommended for preventing or treating female sexual dysfunction among women living with FGM
Information and education
BP-4. Information, education and communication (IEC)4 interventions regarding FGM and women’s health should be provided to girls and women living with any type of FGM
BP-5. Health education5 information on deinfibulation should be provided to girls and women living with type III FGM
BP-6. Health-care providers have the responsibility to convey accurate and clear information, using language and methods that can be readily understood by clients
BP-7. Information regarding different types of FGM and the associated respective immediate and long-term health risks should be provided to health-care providers who care for girls and women living with FGM
BP-8. Information about FGM delivered to health workers should clearly convey the message that medicalization is unacceptable

For further information on how the determinations between a recommendation and a best practice statement were made, see WHO 2016 Guidelines [15]

International human rights treaties provide an important framework of understanding FGM as a practice that constitutes a violation of human rights, especially those of women and girls. They address the impact of FGM in hampering women and girls’ exercise and enjoyment of human rights and gender equality. At the same time, they provide guidance about appropriate responses to FGM by health providers, particularly in the jurisprudence established in Treaty Monitoring Bodies’ (TMBs’) general recommendations and comments. TMBs are committees of independent experts that monitor implementation of the core international human rights treaties. As such, international human rights norms and standards give guidance covering governments’ obligations at a legislative and policy level and specific obligations and appropriate actions of health providers, either as state or non-state actors [19]. Many regional human rights agreements, national policies, and state/provincial policies also take up the issue of FGM, though we focus here on international and national policies and efforts.

## Human Rights Standards in relation to FGM

WHO Guidelines (Table 1) underscore the fundamental importance of providing treatment and care to women and girls survivors of FGM and have stated “Girls and women living with female genital mutilation have experienced a harmful practice and should be provided quality healthcare.” [15]. The WHO, “as part of its core mandate to provide assistance to Member States in achieving the goal of the highest attainable standard of health for all, issued a 2008 interagency statement… [which] declared vigorous support for its abandonment. The aspiration to alleviate the associated adverse health conditions and to restore violated human rights constitutes the cornerstone of these guidelines.” [3, 15].

TMBs have consistently made clear that harmful practices like FGM constitute a violation of women and girls’ human rights [20] and are a form of **discrimination** based on sex, gender, age and other grounds. (Paragraph 49) [21].

FGM sustains gender norms and stereotypes that contravene human rights, and is harmful to the health and wellbeing of girls and women. A number of international human rights conventions explicitly and implicitly address states’ obligations to eliminate FGM. The Convention on the Elimination of All Forms of Discrimination against Women requires states to “take all appropriate measures, including legislation, to modify or abolish existing laws, regulations, customs and practices which constitute discrimination against women.” (Article 2 (f)).

The Convention on the Rights of the Child (CRC) underscores the importance of ensuing protection and care for children and recognizes the responsibility of state parties in this regard (Article 3). The CRC also established the “best interests of the child” standard in addressing the rights of children (Article 3) as well as autonomy related to their evolving capacity. FGM is recognized as a violation of that best interest standard and a violation of children’s rights. The CRC mandates states to abolish “traditional practices prejudicial to the health of children.” (Article 24 (3)).

CEDAW and CRC Committees have made numerous observations recognizing FGM and other harmful practices as “harmful to the health of women and children” [20, 22, 23] and “carry a high risk of death and disability.” [24] For example, FGM “may have various immediate and/or long-term health consequences, including severe pain, shock, infections and complications during childbirth (affecting both the mother and the child), long-term gynecological problems such as fistula, psychological effects and death.” (Paragraph 19) [22].

The Committees have underscored the key role that health providers and others working with girls and young women can play in identifying actual or potential victims of FGM and highlighted how confidentiality rules may be incompatible with their obligation to report incidents of FGM [23]. They have recommended that states parties make it “mandatory by law for professionals and institutions working for and with children and women to report actual incidents or the risk of such incidents if they have reasonable grounds to believe that a harmful practice has occurred or may occur.” (Paragraph 55) [23]. The Committees have further recommended that states provide front-line professionals with relevant information and training to be able to respond to incidents of FGM and to provide specialised training to health-care providers working with immigrant communities. (Paragraph 72d) [23].

These findings are further elaborated under the results sections.

## Methods

This review was conducted to explore the international human rights basis for the recommendations made in the Guidelines. These Guidelines provide detail on how health providers can carry them out, including deinfibulation, mental health, female sexual health and information and education. Forthcoming training curricula from the WHO will provide further guidance for both in-service and pre-service capacity building. The starting point of the review was the Inter-agency Statement on Eliminating FGM, which identified several human rights violations experienced by women in relation to FGM [3].

The review of human rights standards was conducted to cover reports, concluding observations and general comments of the UN Human Rights Council, Treaty Monitoring Bodies and Special Rapporteur reports. Four databases were searched for the review: the Office of the High Commissioner on Human Rights (OHCHR) Universal Human Rights Index; bayefsky.com; the University of Minnesota Human Rights Library; and the Universal Periodic Review (UPR). Findings included results from documents of the Committee against Torture; Committee on the Elimination of Discrimination against Women; Committee on the Rights of the Child; Committee on Economic, Social and Cultural Rights; Human Rights Committee; Special Rapporteur on Health and Special Rapporteur on Torture. Relevant findings of the UN Human Rights Council, Treaty Monitoring Bodies and Special Rapporteurs (these included reports, concluding observations and general comments) were also reviewed in relation to normative developments regarding FGM. The review was done for findings from 1996–2016 for documents in English.

All findings (these include Concluding Observations, General Comments and Recommendations) where international human rights bodies had *explicitly* made observations on FGM were included, but also those that were implicitly dealing with these issues via a discussion about harmful practices (even if not explicitly referring to FGM). Based on this initial search, data was extracted from these findings and organised according to human rights norms and standards that explicitly addressed FGM, prevention or treatment. Human rights standards that addressed harmful practices for women and girls were included. The findings were synthesized along the emerging themes of violence against women, discrimination, right to health and physical integrity, and right to remedy and accountability. These organising categories are ones that emerged as cross cutting across different human rights. Findings without a specific focus on issues related to FGM or harmful practices were excluded.

## Results

Acknowledging the indivisibility and interconnectedness of human rights, FGM may violate multiple human rights, as reflected in the discussion in this section.

Based on search terms FGM, FGC, and harmful practices, and the inclusion criteria highlighted above, a search of the Universal Human Rights Index for “female genital” resulted in 400 TMB findings, which includes concluding observations across six TMBs. These results were then categorized into areas of rights violation identified by the TMB. The suggested actions by the TMB fell primarily into five areas, listed below:

1. Pass or strengthen legislation
2. Implement legislation and policy, particularly in increasing prosecutions.
3. Improve data collection
4. Increase awareness-raising and education targeting families, providers and medical personnel, religious authorities, in collaboration with civil society organisations.
5. Establish support mechanisms, including access to justice/remedy for victims.

The TMBs concluding comments and observations have found that FGM violates a range of rights, including, *inter alia,* women’s rights, children’s rights, freedom from discrimination, freedom from violence, the right to health, the prohibition of torture and cruel, inhuman and degrading treatment, rights related to marriage and family, right to an effective remedy, and the right to education and information. The TMB’s concluding comments/observations also highlight the potential unintended consequences of laws, policies and national action plans that are designed to address or include addressing FGM and harmful practices [25].

Across the TMB’s comments, it is clear that the practice is linked to a broad range of issues that contravene human rights obligations. For example, the CEDAW Committee has welcomed awareness-raising campaigns on FGM, but expressed concern “at the persistence of adverse cultural norms, practices and traditions, as well as patriarchal attitudes and deep rooted stereotypes regarding the roles and responsibilities of women and men in the family and society.” It notes that “stereotypes contribute to the persistence of violence against women and harmful practices.” [26]. In its concluding observations the Committee on Economic, Social and Cultural Rights has raised similar concerns, commenting that invoking “traditional values to explain practices that are not in line with obligations flowing from international human rights law, such as polygamy, FGM, as well as corporal punishment of children in schools”, was in violation of the rights under the Covenant [27]. In another report CEDAW further notes that, “stereotypes contribute to the persistence of violence against women as well as harmful practices…” [28].

### Ending gender-based violence (GBV), including harmful traditional practices

FGM itself has often been described as a form of violence against women and girls, as well as a harmful practice and a health issue.

TMBs have highlighted that in crafting legislation, policy and health practitioner guidelines on FGM, it is important to ensure the state does not fail to respect, protect and fulfil rights, either by taking decision making out of the hands of victims/survivors or by overly relying on the punitive policies and actions from the state that result in discrimination against women. For example, in the cases of countries with mandatory prevention laws (for example, ‘duty to report’ clauses in some countries’ FGM legislation that require health providers to report suspected girls at risk of undergoing FGM to the authorities) [30] or in the case of GBV mandatory arrest, both risk the state taking actions that “*thwart[ing] rather than advanc[ing] fundamental human rights principles of safety, equality and dignity.”* [29].

Debates about FGM and criminalization are similar to debates about the criminalization of GBV. TMBs have also raised concerns with regard to increased policing, prosecution and imprisonment or the criminalisation of GBV has been presented as the solution to GBV, but it may well place women at greater risk of state violence [30]. The risk of focusing on the use of the strong arm of the state is that victims of FGM will fear exposing themselves to health providers, utilizing health services only in emergency situations – thereby increasing the danger to themselves and the complexity and urgency of treatment for health providers. Thus, TMBS have highlighted that responses by the state that rely primarily on criminal law and punitive policies risk deterring the very people who are in greatest need of awareness-raising, social and legal support and education, in addition to health services [31].

Social, cultural, community norms in relation to gender often create unique challenges for health providers who are working with women and girls living with FGM. In such a context, TMBs have pointed out that efforts to resist and eradicate FGM require multi-sectoral, gender- and culturally-sensitive response that works across sectors, communities and generations [32].

Accordingly, the WHO Guidelines consider that “while legal prohibitions create an important enabling environment for abandonment efforts, and criminal prosecutions can send a strong message against the practice, if these are not combined with education and community mobilization, they risk placing health-care practitioners in the position of enforcers of punitive policies, potentially damaging their relationships with their clients and limiting their capacity to engage in rights-based and gender-equality-promoting health practices.” [15].

The TMBs consider FGM to be a form of GBV, and more specifically, violence against women. Calls for new or strengthened legislation prohibiting violence against women and harmful traditional practices, including FGM, can be found in many TMB’s concluding observations. This is the case in CEDAW’s concluding comments to Chad’s 2011 periodic report. CEDAW calls on the state party “to provide for sanctions against perpetrators of violence against women, including FGM, early marriages and domestic and sexual violence, and ensure the investigation of cases, as well as the prosecution and punishment of perpetrators.” [33].

### Discrimination against women/women’s social status

While the distinction between GBV and discrimination is not always clear (for example, in CEDAW, violence against women is considered to be a form of discrimination, and therefore, covered by the convention), in some cases the TMBs make special note of FGM within the context of discrimination. For example, in one of their concluding observations, the CEDAW committee calls on a state party “to raise public awareness, through the media and education programmes, of the fact that all forms of violence against women, including FGM, are a form of discrimination under the convention and therefore in violation of women’s rights.” [34].

Several TMBs also raise concerns that state parties’ legislation and policy might foster discrimination. For instance, CERD in its concluding observations highlighted the importance of protecting girls at risk in immigrant diaspora communities that practice FGM through mandatory reporting by health providers and teachers, and the importance of precautionary measures such as withholding passports from families seeking to take underage girls home to their countries of origin, where they suspect FGM will be performed upon them. In this context, the Committee cautioned and expressed *concern that such measures could lead to excessive focus on these issues which may be seen as stigmatizing women and girls belonging to certain minority groups.”* [35] (Emphasis added.) The Committee recommended measures to be taken to protect girls and women against stigma and promote their human rights [36].

### Right to an effective remedy and lack of accountability

The TMBs consistently note the lack of information (inadequate collection of data on cases of FGM) and implementation of legislation (lack of prosecution) as major challenges. For example, in the CCPR’s concluding observations in 2012, they noted the issue of the right to an effective remedy specifically, calling on the state party to ensure that cases of FGM and domestic violence are thoroughly investigated, that perpetrators are brought to justice, and the victims adequately compensated [37].

Questions about accountability are also frequently identified, with many of the treaty bodies commenting on inadequate reporting. For example, the CRC has expressed its concern about the lack of up-to-date information on measures taken by the state parties to prevent and eliminate harmful traditional practices, including progress in the implementation of its earlier recommendations [38]. The CRC also expressed its concern about the “lack of research on the prevalence of FGM” and also calls attention to the lack of knowledge about the law prohibiting FGM, “including by health workers.” [39]. The Committee Against Torture (CAT) raised similar issues, commenting that it remains concerned by the fact that girls continue to be subjected to FGM. CAT also highlighted its concern regarding “the absence of detailed information on the complaints that have been filed and the investigations conducted into those complaints, on the legal proceedings brought against those responsible for this practice and on the penalties imposed upon them.” [40].

### Right to health and physical integrity

Not surprisingly, several TMBs make reference to FGM as a violation of the right to health, sometimes explicitly and often implicitly. The CRCs’ observations provide a good example. The Committee has recommended state parties to strengthen its legislative measures regarding FGM and conduct awareness-raising campaigns to combat and eradicate this and other traditional practices *harmful to the health*, survival and development of children, especially girls [41]. [Emphasis added.] The CESCR has also made specific reference to FGM as a violation of women’s physical integrity, and notes that “despite efforts to combat the practice of female genital mutilation (excision), this practice, which *violates the rights and physical integrity of women*, persists in certain regions of Benin and laws criminalizing female genital mutilation and the law on sexual and reproductive health have not been enforced.” [42] [Emphasis added].

UN TMBs and experts have raised concerns regarding the issue of compulsory gynaecological screening of girls presumed to be at risk. The issue is also being widely debated at the regional level. A recently appointed commission at the European Level questioned whether the governments had the authority to force underage girls to undergo such examination, and, furthermore, the commission noted that it would, in effect, be treating them as perpetrators and not as victims. The commission noted that such requirements would be imposed only on a specific group, thus amounting to discrimination [43].

### Prohibition of Torture and cruel, inhuman or degrading treatment

The CAT has consistently addressed FGM in their concluding comments. The CESCR also covers FGM, and has explicitly refers to the practice as one that “constitutes cruel, inhuman or degrading treatment.” [44]. The CRC has explicitly directed state parties to enact legislation that will abolish the practice of FGM as it is a violation of the rights of children. In cases where the state fails to act with due diligence, the Convention against Torture and Other Cruel, Inhuman, or Degrading Treatment or Punishment may also apply.

### Medicalisation of FGM

Some states, with the intention of reducing the harms associated with FGM, have made efforts to shift the practice from traditional practitioners to health providers within facilities. These efforts may be predicated upon the acceptance of FGM as a cultural practice, or a belief that it will continue to occur regardless of prevention efforts. The Guidelines Development Group underscored the rejection of medicalization on the basis of international consensus that FGM is a human rights violation that should never be practiced. The guiding principle of the WHO Guidelines (Table 1) highlight that medicalization is never acceptable because it “violates medical ethics since (i) FGM is a harmful practice; (ii) medicalization perpetuates FGM; and (iii) the risks of the procedure outweigh any perceived benefit.” [15]. The Guidelines state that “A number of health-care providers still consider certain forms of FGM not to be harmful, and a large proportion of them are unable or unwilling to state a clear position when confronted with issues like requests for performing FGM or re-infibulation… [T]he involvement of health-care providers in performing FGM is likely to confer a sense of legitimacy on the practice and could give the impression that the procedure is good for women’s health, or at least that it is harmless.” [15].

Medicalisation of FGM is an issue dealt with across TMBs. TMBs have expressed concerns about the medicalisation of FGM, (efforts to encourage health providers in facilities to perform FGM instead of traditional practitioners, based on the false premise that this shift would decrease serious health complications) [45]. Some medicalisation policies allow health providers to perform FGM [46] if they deem it to be a so-called “medical necessity,” [47] despite global consensus amongst major international bodies that there are no known health benefits from the practice [15]. TMBs have asked States to repeal these regulations, to implement laws that prohibit FGM and to ensure adequate penalties for its perpetrators [36, 37]. Moreover, “where medical professionals, government employees or civil servants are involved or complicit in carrying out harmful practices, their status and responsibility, including to report, should be seen as an aggravating circumstance in the determination of criminal sanctions or administrative sanctions such as loss of a professional license or termination of contract, which should be preceded by the issuance of warnings.” (Paragraph 50) [23].

The International Covenant on Civil and Political Rights (CCPR) has expressed concern regarding claims that medical FGM will protect women from riskier procedures performed by traditional practitioners [48]. The Committee has expressed serious concerns regarding rise in procedures by medical practitioners and has called for better protection for women [48]. The CESCR raised similar concerns highlighting that despite its bans in different countries, female genital mutilation continues to be widely practiced, including on so-called medical grounds… [49].

## Conclusions

The 2012 UN General Assembly resolution on “Intensifying global efforts for the elimination of female genital mutilations” urged Member states to “pursue a comprehensive, culturally sensitive, systematic approach that incorporates a social perspective and is based on human rights and gender-equality principles in providing education and training to families, local community leaders and members of all professions relevant to the protection and empowerment of women and girls in order to increase awareness of and commitment to the elimination of female genital mutilations;” and “to develop, support and implement comprehensive and integrated strategies for the prevention of female genital mutilations, including the *training of social workers, medical personnel, community and religious leaders and relevant professionals, and to ensure that they provide competent, supportive services and care to women and girls who are at risk of or who have undergone female genital mutilations* and encourage them to report to the appropriate authorities cases in which they believe women or girls are at risk.” [19] [*Emphasis added*].

The analysis above based on UN human rights treaty body concluding comments and observations shows two levels of gaps and challenges. At the first level, TMBs, Special Procedures and others which have consistently been dealing with the issue of FGM, the issue has been dealtwith on an ad hoc basis with certain elements largely around prevention been given greater attention. The second level of gaps and challenges based on examination by TMBs address the inadequacy of implementation, ranging from failing to fully implement and enforce existing laws (for example, the failure of the UK government to prosecute perpetrators until recently), to foreseeing and addressing unintended consequences of laws and policies (ensuring, for example, that laws and policies do not generate stigma in communities in which FGM is practiced, making it more difficult to detect and prevent FGM), to taking actions that may increase the practice, such as the “harm reduction” measure of medicalisation.

All of these concerns about violations— or inadequate protection— of women and girls’ human rights have significant implications for the work of health care providers. Clearly, FGM raises a series of difficult issues for healthcare providers, from a human rights and gender equality perspective in three senses: first, as a violation of human rights in which caregivers have a moral obligation to address and impede; secondly, as an act of violence persons to whom care providers have the obligation to try to prevent; and thirdly as a practice that generates serious long-term health consequences for women and girls living with FGM [41]. UNFPA, in a toolkit for midwives, states the obligation of healthcare providers in clear terms: “Any health care professional who performs FGM is violating girls’ and women’s right to life, right to physical integrity, and right to health. They are also violating the fundamental ethical principle: ‘do no harm’. In most countries, it is also a violation of the law.” [50]. The WHO guidelines reiterate these principles by issuing good practice recommendations alongside clinical practice recommendations, giving health care providers concrete knowledge to put human rights approaches into action in their care of girls and women living with FGM [15].

In all cases, it is critical to ensure that the particular health issues of women and girls who have undergone FGM, as well as ensuring that quality sexual and reproductive health care and services are available, accessible, acceptable and of high quality, in order to ensure that all women and girls can exercise and enjoy the highest attainable standard of health, and to express their sexuality in conditions free from discrimination, coercion and violence [22, 51].

## Abbreviations

CAT: Committee Against Torture
CCPR: Covenant on Civil and Political Rights
CEDAW: Convention on the Elimination of All Forms of Discrimination Against Women
CESCR: Committee on Economic, Social and Cultural Rights
CRC: Convention on the Rights of the Child
EU: European Union
FGM: Female genital mutilation
GBV: Gender-based violence
HIV: Human immunodeficiency virus
OHCHR: Office of the High Commissioner on Human Rights
STI: Sexually transmitted infection
TMB: Treaty monitoring body
UN: United Nations
UNFPA: United Nations Population Fund
UNHCR: United Nations High Commissioner for Refugees
UNICEF: United Nations Children’s Fund
UNSG: United Nations Secretary General
UPR: Universal Periodic Review
WHO: World Health Organization

## References

[1] UNICEF, 2016. Female genital mutilation/cutting: a global concern. Geneva. 2016. http://data.unicef.org/resources/female-genital-mutilation-cutting-a-global-concern.html, Accessed 26 Apr 2016.

[2] UNICEF. Female genital mutilation/cutting: a statistical overview and exploration of the dynamics of change. New York. 2013. http://data.unicef.org/resources/female-genital-mutilation-cutting-a-statistical-overview-and-exploration-of-the-dynamics-of-change, Accessed 26 Apr 2016

[3] WHO Eliminating female genital mutilation: an interagency statement. UNAIDS, UNDP, UNECA, UNESCO, UNFPA, UNHCR, UNHCHR, UNICEF, UNIFEM, WHO. 2008. http://www.un.org/womenwatch/daw/csw/csw52/statements_missions/Interagency_Statement_on_Eliminating_FGM.pdf, Accessed 8 Aug 2015.

[4] Foulcroy JL (2006). Customs, culture and tradition— what role do they play in a woman’s sexuality?. J Sex Med.

[5] Barstow DG (1999). Female Genital Mutilation: The Penultimate Gender Abuse. Child Abuse & Neglect.

[6] Odeku K, et al. Female genital mutilation: a human rights perspective. J Psychol Africa. 2014.

[7] Avalos L, Farrell N, Stellato R & Werner M. Ending female genital mutilation & child marriage in Tanzania. Fordham Int Law J. 2015.

[8] Wright J. Female genital mutilation: an overview. J Adv Nurs. 1996. 10.1046/j.1365-2648.1996.01934.x 8858427

[9] Whitehorn J, Oyorinde O, and Maingay S. Female genital mutilation: cultural and psychological implications. Sex Relat Ther. 2002.

[10] Hearst AA, Molnar MM. Female genital cutting: an evidence-based approach to clinical management for the primary care physician. Mayo Clin Proc. 2013. 10.1016/j.mayocp.2013.04.004 23726401

[11] Barrett H. Female genital cutting: crossing borders. Geography. 2014.

[12] Jones SD, Ehiri J, Anyanwu E. Female genital mutilation in developing countries: an agenda for public health response. Eur J Obstet Gynecol. 2004. 10.1016/j.ejogrb.2004.06.013 15358454

[13] Shell-Duncan B. The medicalization of female “circumcision: harm reduction or promotion of a dangerous practice? Soc Sci Med. 2001. 10.1016/s0277-9536(00)00208-2 11266046

[14] Nour NM. Female genital cutting: clinical and cultural guidelines. Obstet Gynecol Surv. 2004. 10.1097/01.ogx.0000118939.19371.af 15024227

[15] WHO. Guidelines on Management of Health Complications from Female Genital Mutilation. Geneva; 2016. 27359024

[16] UN Resolution adopted by the General Assembly on 25 September 2015 [without reference to a Main Committee (A/70/L.1)] 70/1. Transforming our world: the 2030 Agenda for Sustainable Development. http://www.un.org/sustainabledevelopment/sustainable-development-goals/ Accessed 17 Dec 2016.

[17] Every Woman, Every Child (2015). UN Secretary General’s Global Strategy on Women’s, Children’s and Adolescents Health.

[18] UNFPA-UNICEF Joint Programme on FGM/C. http://www.unfpa.org/joint-programme-female-genital-mutilationcutting. Accessed 21 Dec 2016.

[19] UN General Assembly. “Intensifying global efforts for the elimination of female genital mutilations,” UN GA, A/C.3/67/L.21/Rev.1, 16 November, 2012.

[20] UN Committee on the Rights of the Child, General Comment 15, para. 9, 2013.

[21] UN Joint Comment for Committee on the Elimination of Discrimination against Women, General Comment 31 and the Committee on the Rights of the Child on harmful practices, General Comment 18, paras. 7 and 15, 2014.

[22] UN Committee on the Elimination of Discrimination against Women, General Recommendation 19, para. 20, 1992.

[23] UN Joint general recommendation/general comment No. 31 of the Committee on the Elimination of Discrimination Against Women and No. 18 of the Committee on the Rights of the Child on harmful practices. CEDAW/C/GC/31-CRC/C/GC18. 2014.

[24] UN Committee on the Elimination of Discrimination against Women, General Recommendation 24, para. 12.b, 1999.

[25] UN Office of the High Commissioner for Human Rights. CESCR General Comment No. 14: The Right to the Highest Attainable Standard of Health (Art. 12). E/C.12/2000/4. 2000.

[26] UN Committee on the Elimination of Discrimination Against Women, Concluding Observations, Togo, 20, UN Doc. CEDAW/C/TGO/CO/6-7, 2012.

[27] UN Committee on Economic, Social and Cultural Rights Concluding observations on the initial to third report of the United Republic of Tanzania. UN Doc. E/C.12/TZA/CO/1-3, 2012.

[28] UN Committee on the Elimination of Discrimination against Women. Concluding observations on the sixth periodic report of Angola, para 17. UN Doc. CEDAW/C/AGO/CO/6, 2013

[29] UN Special Rapporteur on Violence Against Women. Report of the Special Rapporteur on Violence Against Women, Addendum: Mission to Azerbaijan. Geneva: UN Human Rights Council. Retrieved from A/HRC/26/38/Add.3, 2014.

[30] Goldschield J, Liebowitz DJ. Due diligence and gender violence: parsing its power and its perils. Cornell Int Law J. 2015;48.

[31] Kaplan-Marcusan A (2009). Perception of primary health professionals about Female Genital Mutilation: from healthcare to cultural competence. BMC Health Serv Res.

[32] UNICEF (2005). Innocenti Digest on FGM/C: Changing a harmful social condition.

[33] UN Committee on the Elimination of Discrimination against Women. Concluding observations, Chad, para 23a, UN Doc. CEDAW/C/TCD/CO/1-4, 4 November 2011.

[34] UN Committee on the Elimination of Discrimination against Women. Concluding observations. Norway, 31, UN Doc. CEDAW/C/NOR/CO/8, 2012.

[35] UN Committee on the Elimination of Racial Discrimination. Report of the Committee on the Elimination of Racial Discrimination Norway, 15, UN Doc. CERD/C/SR.2084, 2011.

[36] UN Committee on the Elimination of Discrimination against Women. Concluding observations. Norway, CEDAW/C/NOR/Q/8/Add.1, 2012.

[37] UN Human Rights Committee. Consideration of reports submitted by States parties under article 40 of the Covenant. Kenya, 15, UN Doc. CCPR/C/KEN/CO/3. 2012.

[38] UN Committee on the Rights of the Child. Concluding Observations. Nigeria, UN Doc. CRC/C/NGA/CO/3-4, 2010.

[39] UN Committee on the Rights of the Child. Concluding Observations. Austria, 37, UN Doc. CRC/C/AUT/CO/3-4, 2012.

[40] UN Committee Against Torture. Concluding Observations. Gabon, 21, UN Doc. CAT/C/GAB/CO/1, 2013.

[41] UN Committee on the Rights of the Child. Considerations of Reports Submitted by States Parties. United Republic of Tanzania, 51, UN Doc. CRC/C/TZA/CO/2. 2006.

[42] UN Committee on Economic, Social and Cultural Rights. Concluding Observations. Benin, 26, UN Doc. E/C.12/BEN/CO/2. 2008.

[43] Commission Fight Against Female Genital Mutilation. Bestrijding Vrouwelijke GenitaleVerminking. Beleidsadvies. Advies uitgebracht door de Commissie Bestrijding Vrouwelijke Genitale Verminking aan de Minister van Volksgezondheid, Welzijn en Sport, Zoetermeer, cited in Leye and Sabbe at note 32. 2005. Accessed 7 Jan 2017. http://www.pharos.nl/documents/doc/rvz2005.pdf.

[44] UN Committee on Economic Social and Cultural Rights. Concluding Observations. Chad, 19, UN Doc. E/C.12/TCD/CO/3. 2009.

[45] UN Committee on the Elimination of Discrimination against Women. Concluding observations. Indonesia, 20, UN Doc. CEDAW/C/IDN/CO/5. 2007.

[46] UN Human Rights Committee. Concluding Observations. Indonesia, 12, UN Doc. CCPR/C/IDN/CO/1. 2013.

[47] UN Committee on the Elimination of Discrimination against Women. Concluding observations. Egypt, 41, UN Doc. CEDAW/C/EGY/CO/7. 2010.

[48] UN Human Rights Committee. Concluding Observations. Indonesia, UN Doc. CCPR/C/IDN/CO/1, 2013.

[49] UN Committee on Economic, Social and Cultural Rights. Concluding Observations. Egypt, UN Doc. E/C.12/EGY/CO/2-4. 2013.

[50] UNFPA. Engaging midwives in the Global Campaign to End Female Genital Mutilation: Toolkit. 2014. Accessed 7 Jan 2017. https://www.unfpa.org/sites/default/files/resource-pdf/TOOLKIT.pdf.

[51] UN International Covenant on Economic, Social and Cultural Rights. General Comment 14. E/C.12/2000/4. 2000.

